# Shear Bond Strength and Enamel Effects of Bioactive Composite Attachments for Clear Aligners

**DOI:** 10.4317/jced.63193

**Published:** 2025-10-17

**Authors:** Thanachot Sirichamratsakul, Wikanda Khemaleelakul, Supassara Sirabanchongkran

**Affiliations:** 1 Department of Orthodontics and Pediatric Dentistry, Faculty of Dentistry, Chiang Mai University, Chiang Mai, Thailand

## Abstract

**Background:**

Attachments improve retention and facilitate complex tooth movement during clear aligner therapy. However, enamel demineralization and white spot lesions remain concerns, particularly in patients with poor oral hygiene. Fluoride-releasing materials help prevent white spot lesions, and bioactive composites, which release fluoride ions, show promise in preventing demineralization while maintaining strong mechanical properties. However, their application in clear aligner therapy remains underexplored. Therefore, this study investigated the shear bond strength (SBS) of three materials-Filtek Z350 XT Flowable (resin composite), Beautifil Injectable X (giomer), and Cention N (bioactive composite) used as attachments in clear aligner therapy under non-thermocycling (T0) and thermocycling (T1) conditions.

**Material and Methods:**

A total of 120 intact maxillary first premolars were randomly divided into three groups according to the material, with each further subdivided into T0 and T1 subgroups. The thermocycling protocol involved 1,000 cycles between 5°C and 55°C to simulate intraoral aging. SBS was tested using a universal testing machine, and failure modes were analyzed with the adhesive remnant index (ARI).

**Results:**

Cention N exhibited the highest SBS (29.783 ± 4.741 MPa), followed by Filtek Z350XT Flowable (23.834 ± 4.708 MPa), while Beautifil Injectable X had the lowest (15.332 ± 4.087 MPa). The SBS was slightly higher in the T0 subgroup than in the T1 subgroup, but the difference was not significant. ARI analysis showed that Cention N was more likely to cause cohesive enamel failure during attachment detachment.

**Conclusions:**

All three materials demonstrated adequate SBS for use as attachments in clear aligner therapy. However, material selection should consider both SBS and potential risks to enamel integrity. These findings provide valuable data for optimizing material choices in clear aligner therapy and highlight the need for further research to assess long-term performance.

## Introduction

Clear aligners have become a popular alternative to conventional fixed appliances due to their aesthetic appeal and comfort. Clear aligners are designed to progressively move teeth using a series of custom-fabricated trays, supported by digital planning tools, to enhance the accuracy of tooth movement while reducing the clinical visits and emergencies often associated with conventional fixed appliances (1). Typically made of resin composites, attachments are essential components of clear aligner therapy, enhancing aligner retention and enabling the application of complex orthodontic forces to achieve challenging tooth movements (2). Despite their effectiveness, the durability of attachments is a critical concern, as bond failure can lead to treatment delays, additional appointments, and potential disruptions to planned outcomes (1). Therefore, attachment materials must combine a high shear bond strength (SBS) with durability and enamel preservation upon detachment. While resin composites remain the material of choice for attachments due to their ease of use, high bond strength, and aesthetic appeal, their lack of bioactivity limits their ability to prevent enamel demineralization during prolonged orthodontic treatment. This issue is particularly relevant as attachments may contribute to enamel surface changes, including white spot lesions (WSLs), in patients with insufficient oral hygiene. WSLs affect a substantial proportion of orthodontic patients, with some studies reporting incidences up to 45.8% (3), with the maxillary anterior teeth being particularly susceptible, especially during fixed orthodontic treatment. However, clinical evidence regarding the occurrence of WSLs during clear aligner therapy remains unclear. Bisht et al. have found that WSLs occur similarly with clear aligners, self-ligating brackets, and conventional brackets (4). Fluoride-releasing materials have emerged as a promising solution to mitigate the formation of WSLs. Conventional fluoride-releasing materials, such as glass ionomer cement (GIC) and resin-modified GIC (RMGIC), have demonstrated efficacy in reducing WSLs. However, they are often limited by lower mechanical properties, making them unsuitable for attachment applications (5). To overcome these limitations, bioactive composites such as Cention N have been proposed as promising alternatives. These materials release ions, especially fluoride, that support enamel remineralization and prevent caries (5). Bioactive composites also exhibit strong mechanical properties, including compressive strength, tensile strength, and SBS comparable to resin composites and superior to RMGICs. These materials also provide sustained fluoride release, further enhancing their anticariogenic benefits (6 , 7). Despite these advantages, the application of Cention N as an attachment material in clear aligner therapy has not been extensively investigated, particularly regarding its SBS and potential effects on enamel integrity during attachment detachment. Therefore, this study aimed to compare the SBS of Cention N to two materials: Filtek Z350XT Flowable, a resin composite, and Beautifil Injectable X, a giomer. It assessed the durability and overall suitability of these materials for orthodontic applications using a thermocycling protocol that simulates intraoral aging conditions. It also investigated failure modes and utilized scanning electron microscopy (SEM) to analyze their effects on enamel integrity and clinical performance. These results are expected to offer valuable insights into the optimization of attachment materials, contributing to more effective and safe clear aligner therapy.

## Material and Methods

This experimental comparative study consisted of 120 intact human upper premolars recently extracted for orthodontic treatment purposes without abnormalities, prior treatment with orthodontic bracket or attachment, buccal restoration, large caries, and pulp necrosis. All specimens were evaluated under a stereomicroscope before the experiment. The materials used in this study are shown in Table 1.

Table 1

**Figure 1:**
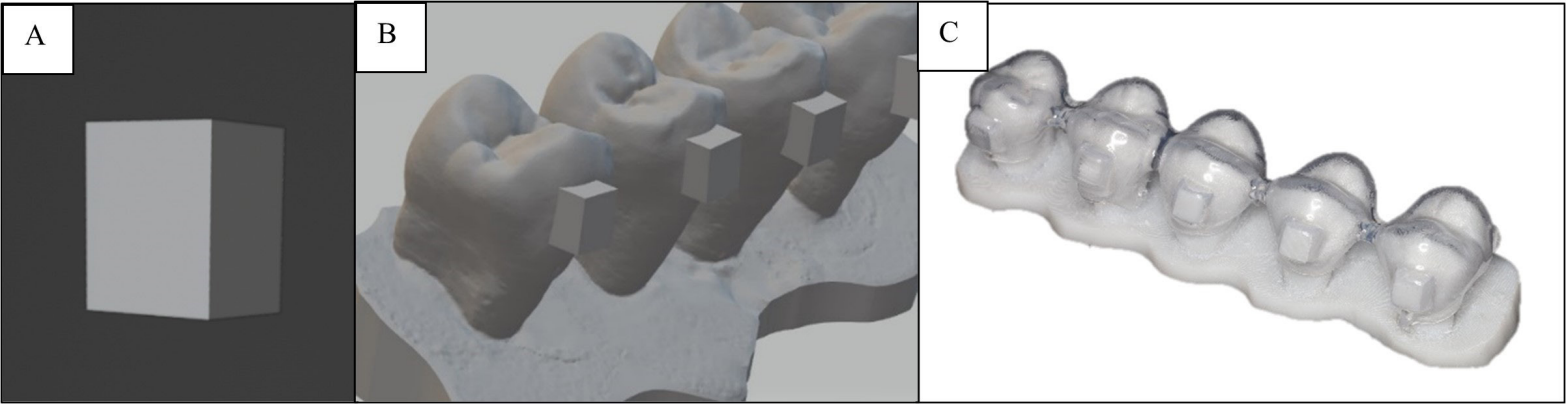
A) the STL file of attachment (rectangular box size of 2.5 x 3.0 x 2.0 mm). B) the STL file of the attachment merged with premolars. C) the attachment template fabricated from the printed model.

- Specimen preparation The extracted premolars were cleaned with water to remove any remaining connective tissues and then stored in a 10% formalin solution. A total of 120 intact premolars were randomly selected and divided equally into three groups according to the material, with each further subdivided into two subgroups according to thermocycling status (without [T0] and with [T1], n = 20 per subgroup). Groups of five teeth were fixed in plaster of Paris to create a stable model and then scanned using an intraoral scanner (TRIOS®; 3SHAPE, Copenhagen, Denmark). - Fabrication of clear aligner attachments and templates A rectangular box measuring 2.5 × 3.0 × 2.0 mm was chosen as the shape of the attachment. The attachment was designed using Blender 3.1 software, merged with the scanned model, and then printed as a 3D model using a Form2 3D printer (Formlabs, Somerville, MA, USA) (Fig. 1).

1

**Figure 2:**
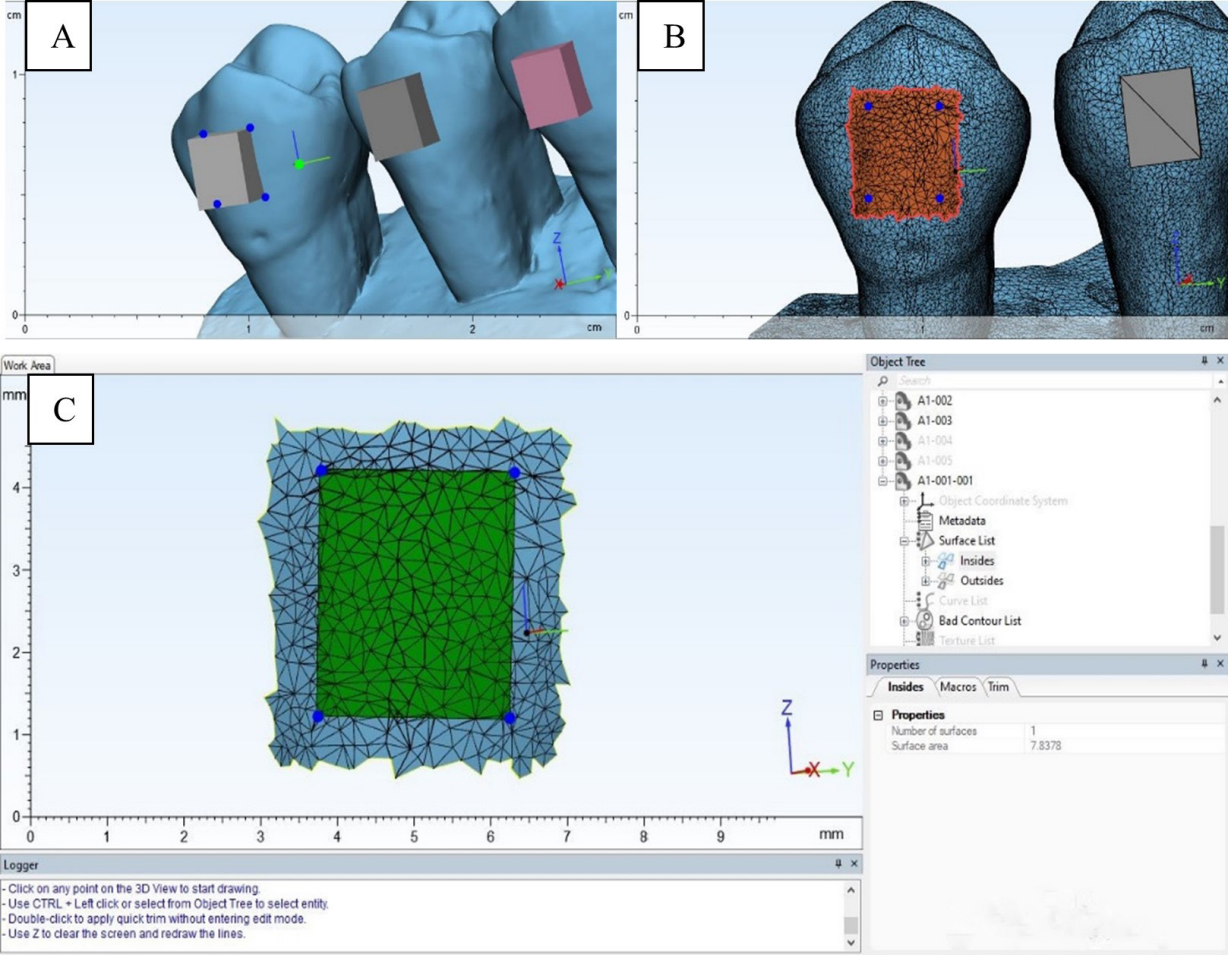
A) separation of the attachment and the tooth by points marked at the angles of the contact point between tooth surface and attachment. B) rectangular marks cover the areas of enamel surface under attachment base. C) the enamel surface area under the attachment was trimmed and calculated.

- Attachment bonding procedure and thermocycling Filtek Z350 XT Flowable (F): Etching with 37% phosphoric acid for 15 seconds, rinsing, and drying, followed by applying the Adper Single Bond 2 adhesive and light curing for 10 seconds before composite application and final curing. Beautifil Injectable X (B): Application of BeautiBond Xtreme self-etch adhesive and leave undisturbed for 10 seconds, followed by air drying and light curing for 10 seconds before material placement and final curing. Cention N (C): Etching with 37% phosphoric acid for 15 seconds, rinsing, and drying. Mixed at a 1:1 powder-to-liquid ratio, load to attachment template, followed by light curing for 20 seconds. Light curing was performed using a high-power light-emitting diode curing unit (Mini LED; Satelec® Acteon Group, Merignac, France) with a 7.5 mm diameter tip and a light intensity of 1,250 mW/cm². The specimens in each group were divided into T0 and T1 subgroups (n = 20 per subgroup). The T1 subgroup underwent 1,000 thermocycling using a thermocycling machine (hot water bath [HWB332R], cold water bath [CWB332R], and temperature sensor [TC301]; King Mongkut's Institute of Technology, Ladkrabang, Thailand) between 5°C and 55°C with a dwell time of 20 seconds and a transfer time of 10 seconds. - SBS test All samples were sectioned using carborundum discs, cutting 2 mm apically from the cementoenamel junction, and embedded in polyvinyl chloride molds filled with orthodontic plaster, ensuring that only the buccal surface of the crown was exposed. Next, the occlusal and gingival surfaces of the attachment were oriented perpendicular to the base of the customized loop-wire simulation fixture. The SBS was tested using a universal testing machine (Instron 5566; Instron Calibration Laboratory, Canton, MA, USA) at a crosshead speed of 1 mm/min, with orthodontic-looped wire simulation. A load was applied in the gingival-occlusal direction until detachment occurred. The results are reported as the loading force (F, newtons [N]). - Enamel surface area calculation The surface area (A, mm2) of the enamel in contact with the attachment was calculated from the STL files (Fig. 2) using the 3-matic Research software (version 13.0; Materialise, Leuven, Belgium).

2

The SBS was recorded in N and calculated using the following formula: SBS (MPa) = F (N) / A (mm2). - Evaluation mode of failure After the attachment was detached, all specimens were inspected under a stereomicroscope (culture microscope [CK40] and digital camera [DP12]; Olympus, Tokyo, Japan) at 20× magnification to evaluate the adhesive remnant index (ARI). The ARI was evaluated using criteria modified from those proposed by Leódido et al. in 2012 (8): 1 = Cohesive failures in enamel. 2 = Interfacial failure between attachment material and enamel. 3 = Bonding areas are partially covered by attachment material &lt; 50% 4 = Bonding areas are partially covered by attachment material 50% 5 = Cohesive failures in attachment material; 100% of bonding areas are covered by composite resin. - SEM observation After detachment of the attachments, the specimens were examined using SEM (fourth generation VEGA; TESCAN, Brno, Czech Republic). To ensure surface cleanliness, the specimens underwent ultrasonic cleaning to remove any residual debris, followed by dehydration via an ascending ethanol concentration. Next, the specimens were left to dry for 48 hours in a sealed environment to minimize external contamination. Then, their surface was sputter-coated with a thin layer of gold using a sputter coater (model 108; Cressington Scientific Instruments Ltd., Watford, United Kingdom) to optimize conductivity. Finally, high-resolution SEM images were taken at 10,000× magnification, allowing for a descriptive evaluation of their surface characteristics. - Statistical analysis The data were analyzed using IBM SPSS Statistics (version 29.0.2.0(20); IBM Corp., Armonk, NY, USA). The normality of each variable was assessed using the Shapiro-Wilk test. The SBS is described using the mean and standard deviation (SD). The effect of different materials on the SBS with and without thermocycling was assessed using a two-way analysis of variance (ANOVA) followed by post-hoc pairwise Tukey's honestly significant difference (HSD) tests if significant (P &lt; 0.05). The ARI was analyzed to compare failure modes, with the findings summarized using descriptive statistics. The SEM images were examined and described based on visual observations.

## Results

The SBS followed a normal distribution in all material groups, and its mean and standard deviation in each group without (T0) and with (T1) thermocycling are presented in Table 2.

Table 2

**Figure 3:**
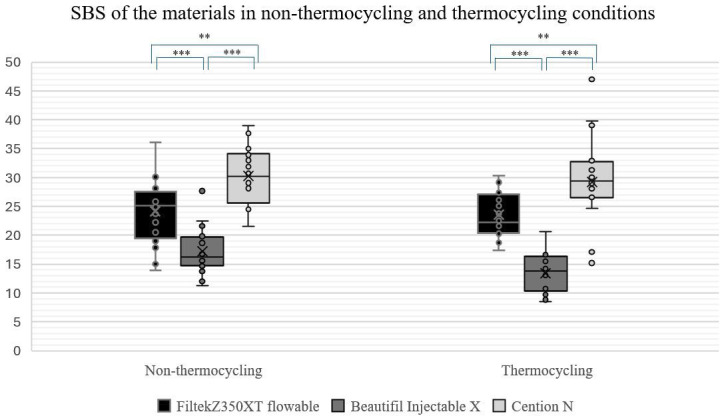
The box plot chart compares the differences of SBS among material groups in the non-thermocycling and thermocycling conditions. Statistical significance is denoted as follows: *P < 0.05, **P < 0.01, and ***P < 0.001.

Two-way ANOVA showed the mean SBS did not differ significantly between T0 and T1 in each material group (P &gt; 0.05). Two-way ANOVA and post hoc Tukey's test showed the mean SBS differed significantly among material groups (P &lt; 0.05) with Group C exhibiting the highest SBS, followed by Group F, while Group B exhibited the lowest SBS for both T0 and T1 (Fig. 3).

3

The ARI scores after bond failure in each group are presented in Table 3.

Table 3

**Figure 4:**
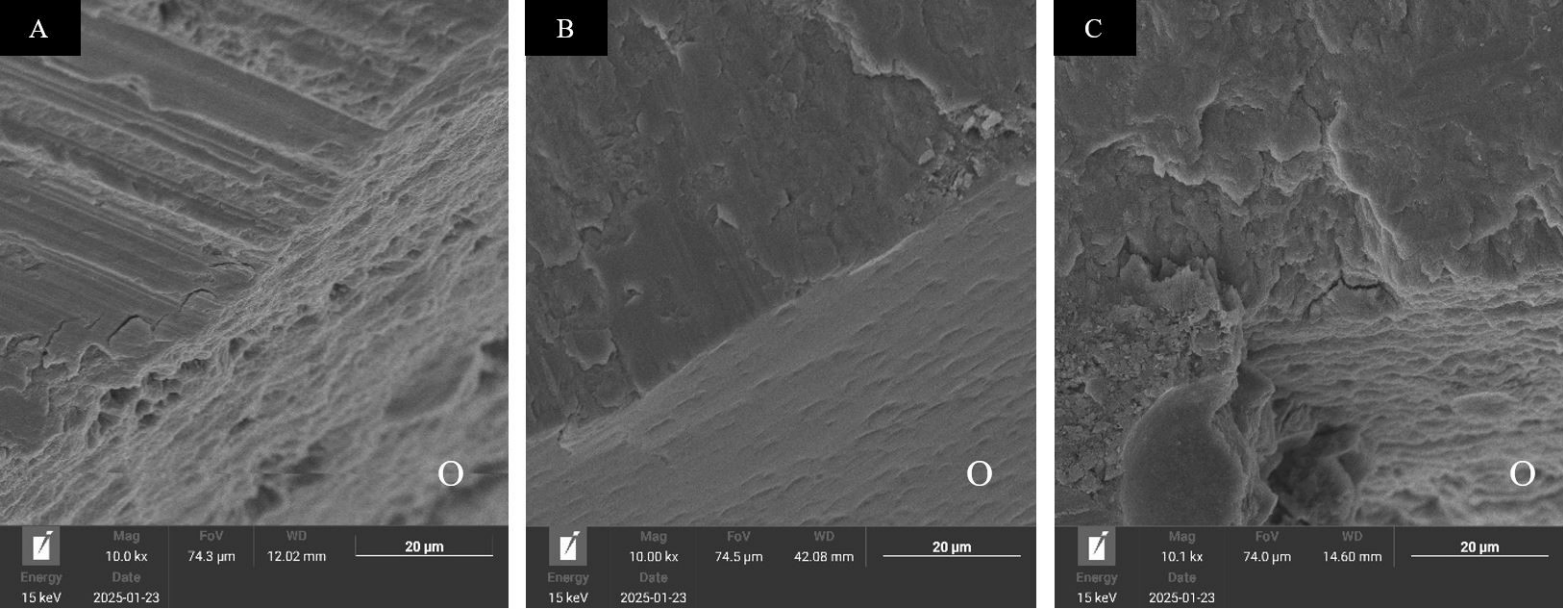
SEM images of enamel microstructure at 10,000x magnification (O indicates the outermost enamel surface side), A: Filtek Z350XT flowable, B: Beautifil Injectable X, C: Cention N.

For both T0 and T1, Group F predominantly exhibited ARI scores of 3 and 4, indicating bond failure with Bonding areas are partially covered by attachment material &lt;50% and 50%. Similarly, Group B primarily exhibited ARI score of 3, indicating bond failure with partially covered by remnant material &lt;50% remnant material. In contrast, Group C demonstrated the highest frequency of cohesive enamel failure. Most specimens in Group C had an ARI score of 2 for T0, indicating interfacial failure between the material and enamel. However, most specimens in Group C had an ARI score of 3 for T1, indicating failure with partially covered by remnant material &lt;50%. The enamel surface of specimens with ARI scores of 2-3 was examined under SEM after the detachment of attachments from intact enamel surfaces. The examination revealed notable differences in microstructure among the three groups (Fig. 4).

4

Group C exhibited high surface irregularity, characterized by areas of partial enamel fracture at the outermost layer (Fig. 4C). In contrast, Group B exhibited a smooth surface with minimal enamel loss (Fig. 4B). Group F exhibited an intermediate surface morphology, with moderate irregularities and less enamel fracture than Group C (Fig. 4A).

## Discussion

The attachment is a crucial part of the clear aligner, enhancing retention and allowing complex tooth movement. Generally, the attachment can be created from resin composite due to its mechanical properties and aesthetic appeal. However, adhesive bond failure can result in the detachment of orthodontic attachments from the tooth surface, potentially leading to significant clinical complications, including extended treatment duration, increased appointment frequency, deviations in planned tooth movement, and potential adverse effects on the overall treatment outcome (9). - SBS of different attachment materials Our study evaluated the SBS of three materials: Filtek Z350XT Flowable (resin composite, Group F), Beautifil Injectable X (giomer, Group B), and Cention N (bioactive composite, Group C). Our findings showed that Cention N exhibited the highest SBS, maintaining superior SBS even after thermocycling. This observation suggests that Cention N provides strong and reliable SBS, making it a promising choice for clear aligner attachments. In comparison, Filtek Z350XT Flowable showed a high SBS, consistent with the results of other studies (10 , 11), but it was lower than that of Cention N. However, Beautifil Injectable X exhibited the lowest SBS, indicating that despite its bioactive benefits, it may compromise the adhesive strength necessary for secure attachment retention during clear aligner therapy. The force needed to remove a clear aligner from teeth with attachments is a crucial factor in ensuring both patient comfort and treatment efficacy. Takara et al. analyzed the removal forces of clear aligners from dental models with vertical rectangular attachments, revealing that the removal force was 3163 ± 378 gf, which corresponds to approximately 31.018 ± 3.717 N (12). Based on this force and assuming an attachment surface area of 6 mm², the estimated SBS per attachment was approximately 5.170 ± 0.620 MPa. These results emphasize the need for attachment materials to provide an adequate SBS, ensuring that they remain securely bonded to the enamel while withstanding the forces exerted during aligner removal. The mechanical properties of dental materials are influenced by their inorganic filler content. Our study found that Cention N exhibited the highest SBS among the tested materials, which may be attributed to its higher inorganic filler load of 78.4% by weight (%wt) (13) than Filtek Z350XT Flowable (65%wt) and Beautifil Injectable X (64%wt) (11 , 14). Jedliski et al. support this observation, concluding that an increase in inorganic filler content correlated with greater SBS in attachment materials (15). A greater filler load generally contributes to improved mechanical properties and stronger adhesion, reinforcing the stability of orthodontic attachments. Cention N is classified as a self-adhesive bulk-fill material that does not require a bonding agent for adhesion. However, François et al. demonstrated that Cention N exhibited significantly greater SBS on dentin surfaces with than without an adhesive system (16). Interestingly, while adhesive systems can enhance the bond strength of bioactive composites, their use with Cention N was associated with reduced inhibition of caries on adjacent enamel compared to non-adhesive applications (17). Several studies have reinforced that etching with 37% phosphoric acid significantly enhances the SBS of self-adhesive materials to enamel surfaces (18 , 19). Therefore, while Cention N can function as a self-adhesive material, pretreatment with etching may be necessary to enhance its performance in clear aligner attachment (20). Notably, no studies have yet investigated the SBS of Cention N on intact enamel surfaces. The SBS of Cention N was greater in our study than in previous studies that did not use adhesive or etchant (6 , 21 , 22). This improvement may be attributed to pre-etching the enamel surface with 37% phosphoric acid, which has been observed to enhance the SBS of Cention N. Acid etching creates a porous enamel surface, facilitating better infiltration of adhesive materials and resulting in stronger micromechanical bonds (23). Cention N exhibited low SBS without surface preparation, which may not be suitable for use as an attachment (24). BeautiBond Xtreme, a one-step self-etch adhesive, was used with Beautifil Injectable X. When bonding to intact enamel, self-etch adhesives generally exhibit lower SBS than etch-and-rinse systems (25). However, despite Beautifil Injectable X demonstrating the lowest SBS in our study, its SBS remained sufficient for use as an attachment. A calcium fluorosilicate (alkaline) glass filler in Cention N releases essential ions, especially fluoride, which plays a crucial role in preventing enamel demineralization, supporting remineralization, and exhibiting antibacterial properties that inhibit the growth of cariogenic bacteria. Cention N releases substantially high levels of fluoride ions but less than traditional glass ionomers. It also provides long-term benefits through the release of both fluoride and calcium ions, which support enamel remineralization (26). Giomers gradually release ions over time without exhibiting an initial burst of fluoride release. While their fluoride release is lower than that of conventional GICs, it remains significantly higher than that of compomers and resin composites (27). Regarding operational ease, Filtek Z350XT Flowable and Beautifil Injectable X provide significant benefits. Their user-friendly flowable properties allow for straightforward loading into attachment templates, enabling efficient and precise fabrication of orthodontic attachments. In contrast, Cention N requires the mixing of its powder and liquid components before use, adding a preparatory step and reducing convenience during handling. Its thicker consistency further distinguishes it from the flowable composites, making it less practical for similar clinical applications. - ARI After detachment, each material exhibited distinct failure modes, which have implications for the stability of attachments and enamel integrity. In our study, an ARI score of 5 indicates cohesive failure within the material, which occurs when internal stresses exceed the material's cohesive strength, resulting in its breakdown. This type of failure reflects the material's inherent strength and is generally favorable in bonded joints, as it shows the bond is stronger than the material itself. ARI scores of 3 and 4 indicates mixed failure, a combination of cohesive failure of the material and adhesive or interface failure. An ARI score of 2 represents adhesive or interface failure, occurring either between the material and the adhesive or between the adhesive and the enamel surface, indicating that the interface is weaker than the material and enamel. Conversely, an ARI score of 1 reflects cohesive failure of the enamel, occurring when the material and interface are stronger than the enamel's cohesive strength. In our results, Filtek Z350XT Flowable demonstrated superior SBS with minimal enamel damage, making it reliable for attachment retention during orthodontic treatments. However, Cention N exhibited a more concerning pattern, particularly when cohesive enamel failure (ARI score of 1) was detected. This failure mode indicates that the SBS between the material and the enamel was too strong, resulting in enamel fractures during detachment, which leads to the loss of surface enamel. The risk of enamel damage is influenced by SBS. The attachments were generally removed using burs. This technique, which is independent of the material's bond strength, may cause fewer enamel fractures during the removal process. - SEM observations The enamel surfaces observed under SEM were influenced by the adhesive protocols and material properties used. Cention N-applied with 37% phosphoric acid etching but without a bonding agent-showed highly irregular surface and partial enamel fractures. In contrast, Beautifil Injectable X effectively preserved the enamel surface, resulting in a smooth finish. This outcome is likely attributed to the mild etching effect of the adhesive and minimal enamel surface damage, which correlates with the ARI. Filtek Z350XT Flowable-bonded with a two-step etch-and-rinse system-showed moderate irregularities. The two-step etch-and-rinse approach seemed to provide a good balance between strong adhesion and enamel preservation. Our study focused on evaluating the SBS on unaltered enamel surfaces. Due to the natural curvature of the tooth, the enamel surface beneath the attachment base was larger than the base itself. To ensure accurate measurement, the Materialise 3-Matic Research software was used to calculate the precise enamel surface area beneath the attachment base. All materials tested in our study demonstrated acceptable SBS for use as clear aligner attachments. When combined with appropriate surface preparation, Cention N exhibited a high SBS along with caries-inhibiting and remineralization properties, making it suitable for patients at high risk of caries. However, it is not recommended for areas subjected to heavy occlusal forces, as detachment during function may lead to enamel damage. The Filtek Z350XT Flowable exhibited a high SBS, minimal enamel damage upon detachment, and ease of handling, making it a reliable option for general use, although it lacks bioactive properties. The Beautifil Injectable X provided an acceptable SBS, low enamel damage, and bioactive benefits, making it appropriate for both general patients and those at increased risk of caries. Ultimately, the choice of material can be guided by the clinician's preference and individual patient needs. Our study had a limitation that should be acknowledged. The thermocycling protocol involved 1,000 cycles, which is notably longer than the ISO Standard Protocol 11405 (1994) of 500 cycles for simulating the aging of biomaterials (28). However, 1,000 cycles correspond to approximately 1.2 months of aging (29), which may represent a relatively short-term process. This limited duration resulted in a slight reduction in the SBS. However, the differences between the thermocycling (T1) and non-thermocycling (T0) groups were not significant across all tested materials, indicating only minimal changes in the SBS performance of the attachment materials. Further research is required to examine the long-term effects of thermocycling under conditions that more closely replicate extended treatment durations. It is also essential to explore the impact of different materials, as well as variations in attachment size, shape, and configuration, on the SBS. Moreover, further studies are warranted to directly compare the SBS of etched and non-etched enamel surfaces of bioactive composite to validate our findings. As our experiments were conducted in vitro, randomized controlled split-mouth clinical trials are recommended to validate our findings.

## Conclusions

Firstly, while the bioactive composite's strong mechanical properties offer potential benefits for enhancing enamel health through its bioactive effects, careful consideration is necessary when selecting it for clear aligner therapy, particularly regarding the potential enamel surface loss during detachment. Secondly, SBS did not differ significantly between the non-thermocycling and thermocycling groups for any of the tested materials tested in the short term. Thirdly, under SEM analysis, Cention N exhibited noticeable enamel damage, with an ARI indicating strong bonds causing enamel fractures. In contrast, Beautifil Injectable X preserved the enamel the best, while Filtek Z350XT Flowable balanced bond strength and enamel preservation. Therefore, the choice of adhesive significantly impacts enamel integrity, with cautious detachment crucial to minimize damage, especially with high-strength materials such as Cention N.

## Figures and Tables

**Table 1:** Presents the material used in this study.

Material	Manufacturer/Lot	Composition
1. Cention N	Ivoclar Vivadent, Schaan, LiechtensteinLot: Z05N34Exp: 2025-04-27	Powder: calcium fluorosilicate glass, barium aluminium silicate glass, ytterbium trifluoride, calcium barium aluminium fluorosilicate glass, isofiller, initiator, and pigment 57 v% (78 wt%)Liquid: methacrylate, initiators, additive, and stabilizer
2. Beautifil Injectable X	Shofu, Kyoto, JapanLot: 082202Exp: 2025-07-31	Bis-GMA, Bis-MPEPP TEGDMA, Aluminofluoro-borosilicate glass filler (50-60 wt%), aluminium oxide, colorant
3. FiltekTM Z350XT Flowable	3M ESPE, St. Paul, MN, USALot: 10100202Exp: 2025-04-23	Filler 45 v% (65 wt%) Yttrium fluoride: 0.1 to 5.0 µm, silica: 20 nm, zirconia: 4 to 11 nm, and zirconia/silica clusters of 0.6 to 10 µm with bis-GMA, TEGDMA, and Procrylat K
4. AdperTM Scotchbond Multi-Purpose etchant	3M ESPE, St. Paul, MN, USALot 10253977Exp: 2026-08-21	37% Phosphoric acid.
5. AdperTM Single bond 2	3M ESPE, St. Paul, MN, USALot: 10099993Exp: 2026-03-24	BisGMA, HEMA, dimethacrylates, ethanol, water, silica particle photoinitiator, methacrylate functional copolymer of polyacrylic and polyitaconic acids, polyalkenoic acid
6. Beautibond X treme	Shofu, kyoto, JapanLot 062351Exp: 2025-08-31	Bis-GMA, TEGDMA, Phosphonic acid monomer, Carboxylic acid monomer, Acetone, water, and photo-initiator.

1

**Table 2:** Means and standard deviations of the SBS in the three groups for T0 and T1.

Group	T0		T1		Total	
	N	Mean ± SD (MPa)	N	Mean ± SD (MPa)	N	Mean ± SD (MPa)
F	20	24.153± 5.534a	20	23.515 ± 3.830a	40	23.834 ± 4.708a
B	20	17.207 ± 3.940b	20	13.458 ± 3.373b	40	15.332 ± 4.087b
C	20	30.318 ± 4.741c	20	29.249 ± 7.762c	40	29.783 ± 6.371c

Superscripts (a, b, and c) indicate significant differences between groups (P <0.05); groups with different letters differ significantly from each other.

**Table 3:** Percentages and Frequencies (f) of ARI score of materials after detached in different time points.

Time point	Group	Score 1(f)	Score 2(f)	Score 3(f)	Score 4(f)	Score 5(f)
T0	F	0.00%(0)	7.50%(1.5)	27.50%(5.5)	35.00%(7)	30.00%(6)
	B	0.00%(0)	10.00%(2)	70.00%(14)	17.50%(3.5)	2.50%(0.5)
	C	22.50%(4.5)	50.00%(10)	27.50%(5.5)	0.00%(0)	0.00%(0)
T1	F	5.00%(1)	15.00%(3)	30.00%(6)	30.00%(6)	20.00%(4)
	B	5.00%(1)	40.00%(8)	45.00%(9)	10.00%(2)	0.00%(0)
	C	20.00%(4)	27.50%(5.5)	50.00%(10)	2.50%(0.5)	0.00%(0)

Score 1 = Cohesive failures in enamel. Score 2 = Interfacial failure between attachment material and enamel. Score 3 = Bonding areas are partially covered by attachment material < 50%. Score 4 = Bonding areas are partially covered by attachment material ≥50%. Score 5 = Cohesive failures in attachment material; 100% of bonding areas are covered by composite resin.

## Data Availability

The datasets used and/or analyzed during the current study are available from the corresponding author.
